# Hepatitis B Virus Genotype Influence on Virological and Enzymatic Measures over Time—A Retrospective Longitudinal Cohort Study

**DOI:** 10.3390/jcm12216807

**Published:** 2023-10-27

**Authors:** Alexa Keeshan, Carolina Fernandes da Silva, Alicia Vachon, Elizabeth Giles, Carla Osiowy, Carla Coffin, Curtis L. Cooper

**Affiliations:** 1 Clinical Epidemiology Program, Ottawa Hospital Research Institute, Ottawa, ON K1Y 4E9, Canada; 2 School of Epidemiology and Public Health, University of Ottawa, Ottawa, ON K1N 6N5, Canada; 3 Faculty of Medicine, University of Ottawa, Ottawa, ON K1N 6N5, Canada; 4 Division of Infectious Diseases, Department of Medicine, University of Ottawa, Ottawa, ON K1N 6N5, Canada; 5 National Microbiology Laboratory, Public Health Agency of Canada, Winnipeg, MB R2C 3A9, Canada; 6 Department of Medicine, University of Calgary, Calgary, AB T2N 1N4, Canada

**Keywords:** cirrhosis, liver fibrosis, nucleoside analogues, antiviral therapy

## Abstract

HBV is a hepatotropic virus with multiple genotypes. It is uncertain if specific genotype(s) influence virological measures and/or liver markers over time. It is unclear whether nucleos(t)ide analogue therapy response is influenced by genotype. In this retrospective longitudinal study, we utilized data from The Ottawa Hospital Viral Hepatitis Program (TOHVHP) to evaluate the role of HBV genotype on viral load, liver enzymatic levels, fibrosis progression, and parenchymal inflammation and steatosis over time. HBV DNA, ALT, and AST levels, as well as transient elastography scores for fibrosis (E) and inflammation/steatosis (CAP), were modeled using mixed-effects linear regression. Interaction terms between HBV genotype and time were included to investigate if there was a difference in trends between genotypes. A total of 393 HBV patients infected with genotypes A-E were included. The mean age was 44.4 years, and 56% were male. Asian (50.5%), Black (29.1%), and White (6.4%) patients were well-represented. By multivariate analysis, we found no evidence that the trajectories of these commonly measured viral or liver measures varied over time by HBV genotype in those receiving HBV nucleos(t)ides and in those not on antiviral therapy.

## 1. Introduction

Hepatitis B virus (HBV) is an enveloped hepatotropic DNA virus that chronically infects approximately 296 million people worldwide [1]. HBV is endemic in the Western Pacific region and Africa. In these regions, most cases of HBV are due to vertical transmission [2,3,4]. Horizontal transmission occurring through exposure to blood or bodily fluids from an infected person (e.g., by needles or sexual contact) is also a major contributor to the HBV incidence cases [4,5]. Untreated chronic HBV infection can result in many adverse health outcomes, including liver cirrhosis, liver failure, and hepatocellular carcinoma (HCC) [3,5,6].

Errors in proofreading activity during replication and reverse transcription introduce mutations in the HBV genome and have resulted in the emergence of distinct HBV genotypes based on a nucleotide difference of over 7.5% [3,7,8]. Although it is a point of ongoing debate as to what constitutes a true genotype, there are currently 8 to 10 recognized distinct HBV genotypes (lettered A to J) and over 40 sub-genotypes [2,3,9,10]. The most common genotypes in North America, Western Europe, Africa, and the Indian subcontinent are HBV genotypes A and D. HBV genotype B and C predominate in Southeast Asia, and genotype F predominates in South America [3,9].

Genotypes C and F are well-established to increase the risk for hepatocellular carcinoma [3,11,12]. Responsiveness to interferon-based treatment is also influenced by genotype. Specifically, HDV DNA, hepatitis B surface antigen level, and liver enzyme decline more rapidly in those with genotypes A and B infection than with genotypes C and D [6,13]. However, many key parameters related to HBV, including the extent to which HBV genotype impacts the progression of chronic HBV infection and the response to oral antiviral HBV therapy, are uncertain. In this analysis, data from The Ottawa Hospital Viral Hepatitis Program (TOHVHP) based in Ottawa, Canada were utilized to describe HBV patient characteristics by genotype and assess the influence, if any, of genotype on commonly monitored viral, enzymatic, and fibrotic measures over time.

## 2. Materials and Methods

Patient data collected by the TOHVHP from January 2014 to June 2022 (Ottawa Health Science Network Research Ethics Board #2004-196) were retrospectively studied. Consenting HBV patients with known HBV genotypes who had not received prior nucleos(t)ide analogue (NA) treatment were included. Patients with the hepatitis C virus (HCV), human immunodeficiency virus (HIV), or hepatitis D virus (HDV) co-infection were excluded. All HBV genotypings were conducted at the Public Health Agency of Canada National Microbiology Laboratory, as previously described [14,15,16] Briefly, HBV DNA was extracted from the serum by silica capture and amplified using HBVPr134/135 outer primers and HBVPr75/94 nested primers. The resulting amplicon was purified and Sanger-sequenced. ClustalX and BioEdit were used for sequence alignment and trimming. HBV genotype was estimated using the NCBI HBV genotyping tool and BLAST analysis.

Patient demographics at baseline (age, gender, race, immigration, employment, and housing status), liver tests, and HBV viral data at baseline and during follow-up (transient elastography [E] scores in kilopascals (kPa), fibrosis stage [F0–F4], controlled attenuation parameter [CAP] score in decibels per meter (dB/m), alanine aminotransaminase [ALT] U/L, aspartate aminotransaminase [AST] U/L, alpha fetoprotein [AFP] µg/L, HBV DNA IU/mL, HBV e antigen [HBeAg], and HBV e antibody [HBeAb] status) were collected. HBV DNA was suppressed if viral DNA was not detected or was <20 IU/mL. ALT and AST levels were classified as being in the normal range if they were below 63 U/L (ALT) and 29 U/L (AST). Fibrosis stage was classified based on E scores (F0–1: 2–8 kPa, F2: 9–10 kPa, F3: 11–14 kPa, and F4: >14 kPa). Liver parenchymal inflammation and steatosis were assessed by CAP scores. CAP scores below 238 dB/m were classified as normal. AFP results were normalized to account for changes in the assay over time by dividing the values by the reference range cut-off.

Patients were stratified according to treatment status during follow-up at TOHVHP: those who received one or more rounds of NA therapy were allocated to the treatment cohort, and those who did not receive any NA therapy were allocated to the surveillance cohort. Baseline was defined as the date of NA initiation or the date of enrollment to TOHVHP, respectively. In general, HBV antiviral treatment was started based on the American Association for the Study of Liver Disease and Canadian Association for the Study of the Liver guidelines [17,18]. However, patient wishes and availability of reimbursement influenced whether and when treatment was initiated. HBV antiviral choice was based on physician selection, reimbursement criteria, and patient wishes.

HBV DNA, ALT, and AST levels, as well as transient elastography scores for fibrosis and inflammation/steatosis, were the primary outcomes investigated and were modeled over time from the baseline in days using mixed-effects linear regression. In mixed-effects regression, random effects are included, which allow an individual’s intercept and slope to vary relative to the intercepts and slopes of other individuals. This accounts for the autocorrelation that arises during longitudinal data collection between serial observations for the same individual. Time frames for regression models were determined based on data availability and data distribution. Patient data following DNA suppression or liver enzyme normalization were censored. For the treatment cohort, sample collection dates were restricted to capture the appropriate slope of decay following initial NA initiation (where baseline data were included, and the noise following initial normalization was reduced). For example, for HBV DNA models, the *x*-axis was restricted to remove non-informative observations that were available during the pre-baseline period, which preceded the range of linear decay related to treatment initiation. The exposure of interest was the HBV genotype. HBV genotype and time interaction terms were used to evaluate if there were differences in slopes between genotypes. In accordance with the interaction hierarchy principle, main terms for HBV genotype and time were also included. Outcome data were log-adjusted based on non-linear distribution and to facilitate model convergence. When limited data variability prevented model convergence for models, which included both random intercepts and random slopes, random intercepts alone were used. Chi-square and Fisher’s exact tests (with Monte Carlo simulation [n = 1,000,000 samples] to estimate *p*-values for large contingency tables with a prohibitively high computational burden) were used for categorical variables when assumptions were met. Kruskal–Wallis tests were used for statistical comparisons of continuous variables. A two-sided alpha level of 0.05 was used to determine statistical significance (*p* < 0.05).

## 3. Results

Our study sample included 339 patients living with chronic HBV infection comprised of five genotypes (Table 1, Appendix A Table A1). We initially identified 393 HBV patients who met the inclusion criteria after excluding two HDV and four HCV co-infected patients. We removed 23 patients who had a history of prior antiviral therapy or use of unspecified medication to treat their HBV infection at baseline. Twenty-eight patients with unknown race were not included. Due to insufficient sample sizes, which precluded meaningful analyses, one patient with genotype F HBV infection and four indigenous patients (genotype B = 3, C = 1) were also excluded from the analysis.

Fifty-six percent of the 339 patients were male, and the mean age was 44.6 years. Patients infected with HBV genotype B were the oldest at baseline (mean age 48.2 years), and the ones with genotype E infection were the youngest (37.6 years). The cohort was multiracial and included Asian (54.9%), Black (32.5%), and White (12.7%) individuals. Genotypes E and A were most common (46.4%, 45.5%) in Black patients. Genotypes B and C were most common in Asian patients (53.2%, 36.0%). Most patients were immigrants to Canada (94.7%).

Unadjusted baseline HBV DNA, ALT, AST, HBeAg and HBeAb positivity proportions, and CAP score differed by genotype (Table 1). At baseline, genotype B patients had the highest median HBV DNA level (6320 IU/mL). HBV genotype C patients had the highest proportion with positive HBeAg (24.3%), as well as the highest median liver enzyme levels. Median HBV DNA (738 IU/mL) was lowest for genotype D. Genotype E patients had the highest proportion with negative HBeAg (98.0%), as well as the lowest median liver enzyme levels. Patients with HBV genotype A infection had the lowest mean CAP score (216 dB/m). The mean CAP score (256 dB/m) was the highest in genotype D HBV.

**Figure 1:**
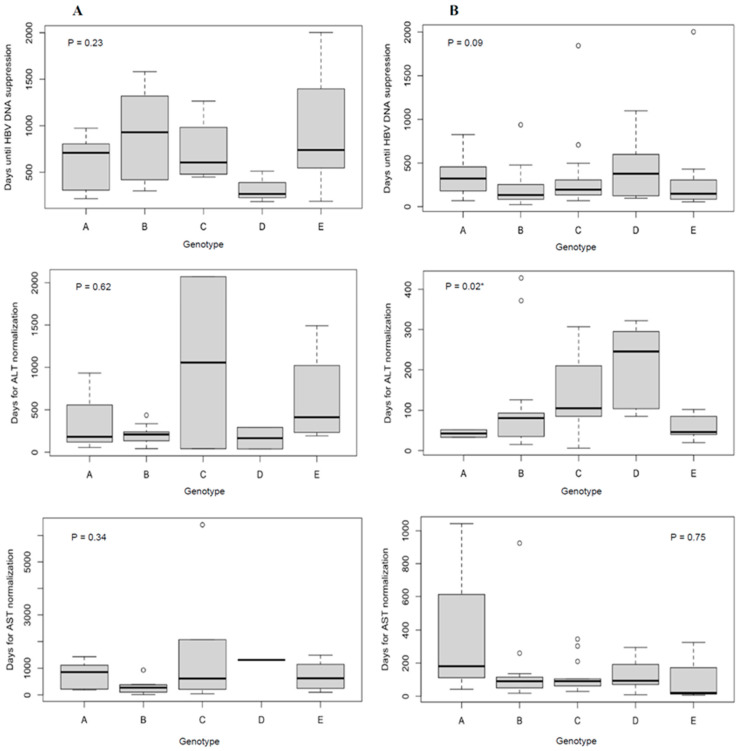
Time to HBV DNA suppression and ALT or AST normalization in days by genotype according to treatment status during follow-up ((**A**) = no treatment, (**B**) = treatment). Note the differing number of days on the y-axis between figures. Outliers are represented by white circles. *p*-values were generated by Kruskal–Wallis tests. * A two-sided alpha level of 0.05 was used to determine statistical significance (*p* < 0.05).

Twenty-eight percent of 339 patients initiated HBV NA antiviral therapy during the follow-up assessment period (Table 1). No patients received interferon-based treatment. For treatment recipients, the crude median time to HBV DNA suppression below the lower limit of quantification was 181 days, and liver enzyme normalization was 91 days (Figure 1). Time to HBV DNA suppression did not differ by genotype. The crude median time to ALT normalization differed by genotype (A = 43 days; B = 81 days; C =105 days; D = 246 days; and E = 46 days; *p* = 0.02).

Regression analysis was conducted to determine if there were differences in HBV DNA, liver enzyme level, transient elastography, and CAP score trends over time according to HBV genotype (Table 2, Appendix A Table A2). There were no interactions between time and genotype and no differences in the adjusted slopes of HBV DNA, ALT, AST levels, CAP score, or fibrosis elastography scores between the different genotypes for those on treatment and those not on treatment. In other words, over time, any changes in the trajectories of these measures did not differ by genotype. Additional models were generated to compare adjusted slopes by HBV genotype for other time frames (up until 365 days after baseline for the untreated cohort and 182 days for the treatment cohort and up until 365 days for both cohorts for the CAP score and the surveillance cohort for E). Similar results were obtained.

Four patients were diagnosed with hepatocellular cancer during follow-up over a median of 8.5 years (Supplementary Table S1). All patients were male, and all received HBV antiviral therapy prior to hepatocellular carcinoma diagnosis. There was a broad and heterogenous range of patient characteristics in terms of genotype (B, B, D, and E), age, fibrosis stage, and duration of HBV antiviral treatment prior to hepatocellular carcinoma diagnosis.

## 4. Discussion

The influence of HBV genotype on the natural history of chronic infection, pathogenesis of liver disease progression, and NA treatment response remains unresolved. This is at least in part due to the geographical distribution of HBV genotypes, which has impeded a fulsome comparison of clinical outcomes between genotypes. Our diverse clinic population addresses this challenge. Some prior analyses suggest that HBV genotype plays a role in the progression of HBV-related liver disease and influences the consequences of HBV antiviral treatment withdrawal, although the mechanism of these influences is yet to be determined [19,20]. In our analysis, we specifically focused on the influence of HBV genotype on HBV viremia and liver enzymes, as well as liver fibrosis and steatosis, cross-sectionally and over time in five high-prevalence HBV genotypes. We did not find evidence that specific genotypes influenced the trajectories of these parameters over time.

Replication dynamics differ across HBV genotypes in vitro, as well as in patient serum studies, which may explain the clinical progression differences that have been reported between genotypes in chronic HBV infection. In our cohort, baseline HBV DNA and liver enzyme levels differed by genotype. Genotype B patients had the highest HBV DNA levels. Genotype C patients had the highest liver enzyme levels and proportion with HBeAg positivity. Our data are consistent with other studies, as genotype C infection has been linked to higher HBeAg-positive status proportions and delayed HbeAg seroconversion, compared to genotype B [21,22,23,24]. These findings are relevant, as high HBV viral loads and HBeAg positivity are associated with higher risks of severe liver disease [23], and high viral load genotype C has been associated with an increased hepatocellular carcinoma risk [25]. Additionally, genotype B has been associated with fulminant hepatitis and acute liver failure in acute infection [26,27]. HBeAg is used clinically as a marker of viral replication, severity of disease, and response to antiviral treatment, due to its dual roles in the activation and modulation of T cell activity in chronic infection [28]. Consequently, HBeAg most likely plays a role in the establishment and persistence of chronic infection [22,28,29].

While there is a clear link between genotypes B and C replication dynamics and clinical outcomes, such as cirrhosis, fibrosis, and fulminant hepatitis, the contributions of other factors, including race, remain to be elucidated. Genotypes B and C are mostly prevalent in people of Asian ethnicity and genotypes A and E in those of Sub-Saharan African origin in Canada [23]. HBV persistence has been attributed to other variables, including mode of transmission, inoculum, and host-factors [25]. The roles of these multiple factors merit further investigation. We found by multivariate analysis that viral levels and liver enzymes were higher with HBV genotypes B and C infection. This suggests that the natural history and high replication phenotype of these genotypes are directly linked to the severity of liver inflammation in chronic infection.

Infection with HBV genotype C is associated with a higher risk of liver fibrosis progression and cirrhosis, but it is unclear if HBV genotypes B, D, and/or F also carry an increased risk of fibrosis advancement [3,5,6,9,11,30,31,32]. In our analysis, there was no apparent genotype influence on the trajectory of HBV DNA, liver enzymes, fibrosis, or inflammatory/steatosis parameters over a multi-year period of observation. HBV genotype B may lead to the development of HCC at a younger age, and HBV genotype C may lead to an increased risk of HCC at an older age [9,10,30,31,33,34]. A low HCC incidence in our cohort precluded the evaluation of HBV genotype and HCC risk. However, it is noteworthy that there was a broad and heterogenous range of patient characteristics, including the extremes of age and fibrosis stage. HCC occurred with multiple different genotypes. This serves as a reminder that all individuals living with chronic HBV infection are at risk for HCC, irrespective of characteristics, and that there are no groups or specific patient profiles that can be exempted from HCC screening guidelines. All four of our HCC patients were on HBV antiviral therapy with suppressed HBV DNA. These medications reduce but do not eliminate HCC risk.

We found that HBV genotype D samples had the lowest median DNA levels, as well as the highest CAP scores. This low HBV DNA level result was unexpected, as genotype D has been recognized as a highly replicative phenotype [35,36] and has been associated with severe liver disease outcomes, such as cirrhosis and HCC, compared to genotype A [3,25]. While HBV genotype has not been associated with the development of steatosis [37], animal models have shown that the presence of metabolic-associated fatty liver disease (MAFLD) in chronic HBV infection may reduce HBV replication, as measured by HBeAg, HBsAg, and HBV DNA levels [38]. It is still unclear whether the concomitant presence of chronic HBV infection and steatosis leads to faster progression to HCC. However, concurrent chronic HBV infection and steatosis or metabolic disorder increase the risk of severe fibrosis [39,40], and consequently, both steatosis and HBV infection require appropriate management to reduce progressive liver disease risk. It is noteworthy that while high HBV viral loads are associated with increased HCC risk [41], there is still a perceivable risk in patients with advanced fibrosis and low HBV viral loads [42]. Thus, NA therapy is recommended to reduce the risk of HCC in these cases.

We assessed the role of HBV genotype on NA treatment response. Our analysis is consistent with most literature suggesting that there is no difference in NA response by genotype based on HBV viral response [10,30,43,44]. Median time to ALT normalization differed between genotypes. However, the clinical relevance of this finding is unclear. Note that we used a relatively high level of aminotransaminase for defining the normalization of liver enzymes.

We reported on a cohort of patients infected with sparsely studied African HBV genotypes, namely A1, A3, and E. These genotypes have been linked to the rapid progression and higher incidence of HCC, in addition to early HBeAg seroconversion [35,45]. Interestingly, genotype E patients had the lowest liver enzymes and were almost all HbeAg negative, which is consistent with previous reports [35,46,47]. We note that the time to ALT normalization after initiating NA treatment was relatively rapid in genotype E, compared to genotypes B, C, and D.

While our analysis has many strengths, including representative cases from five major HBV genotypes, some limitations are recognized. The sample size and length of follow-up may be insufficient to fully elucidate the association between HBV genotype and severe liver disease progression. The sample size in our cohort was insufficient to conduct genotype subtype level analysis. We plan to conduct subsequent analyses focused on genotype subtypes. Longer durations of follow-up may provide additional insights as to the influence of genotype on commonly assessed measures of viral and liver status. The numbers of indigenous patients, genotype F, and HCC cases precluded detailed evaluation. Lastly, this study did not measure the effect of HBV mutations on replication dynamics and clinical outcomes.

## 5. Conclusions

In conclusion, our analysis suggests that HBV NA antiviral treatment response, as assessed by serial viral, enzymatic, and liver elastography measures, is not influenced by genotype. The trajectory of these measures over time in those not receiving HBV antiviral therapy does not differ by genotype.

## Figures and Tables

**Table 1:** Baseline patient demographics and HBV infection characteristics.

Variable		Genotype					*p* ^1^
	Overall	A	B	C	D	E	
	n = 339	n = 63	n = 100	n = 71	n = 52	n = 53	
Gender, n (%) Female	150 (44.3)	24 (38.1)	47 (47.0)	33 (46.5)	18 (34.6)	28 (52.8)	0.29
Male	189 (55.8)	39 (61.9)	53 (53.0)	38 (53.5)	34 (65.4)	25 (47.2)	
Age, mean (SD) Range (min, max)	44.6 (13.2) 60 (17–77)	43.4 (13.6) 52 (18–70)	48.2 (12.7) 54 (23–77)	47.7 (14.3) 58 (19–77)	41.8 (12.3) 51 (17–68)	37.6 (9.5) 41 (21–62)	<0.0001
Race, n (%) White	43 (12.7)	4 (6.4)	1 (1.0)	4 (5.6)	32 (61.5)	2 (3.8)	<0.0001
Black	110 (32.5)	50 (79.4)	0 (0)	0 (0)	9 (17.3)	51 (96.2)	
Asian	186 (54.9)	9 (14.3)	99 (99.0)	67 (94.4)	11 (21.2)	0 (0)	
Immigrated to Canada, n (%)							0.13
Yes	319 (94.7)	60 (95.2)	94 (95.0)	67 (94.4)	46 (88.5)	52 (100.0)	
No	18 (5.3)	3 (4.8)	5 (5.1)	4 (5.6)	6 (11.5)	0 (0)	
Unknown	2	-	1	-	-	1	
HBV DNA (IU/mL), median (IQR)	1810 (3000–47,700)	1190 (206–4840)	6320 (652–82,950)	3480 (300–547,000)	738 (165–83,350)	1330 (165–3520)	0.002
Unknown HBV DNA, n	2	-	-	1	-	1	
HBeAg, n (%) Positive	36 (11.1)	5 (8.2)	9 (9.6)	17 (24.3)	4 (8.2)	1 (2.0)	0.003
Negative	289 (88.9)	56 (91.8)	85 (90.4)	53 (75.7)	45 (91.8)	50 (98.0)	
Unknown/Not tested	14	2	6	1	3	2	
HBeAb, n (%) Positive	283 (87.9)	57 (95.0)	81 (87.1)	51 (72.9)	44 (89.8)	50 (100.0)	<0.0001
Negative	39 (12.1)	3 (5.0)	12 (12.9)	19 (27.1)	5 (10.2)	0 (0)	
Unknown/Not tested	17	3	7	1	3	3	
Fibrosis Stage ^2^ F0–1	245 (87.8)	49 (96.1)	74 (85.1)	46 (80.7)	37 (88.1)	39 (92.9)	0.59
F2	13 (4.7)	0 (0)	5 (5.8)	5 (8.8)	2 (4.8)	1 (2.4)	
F3	12 (4.3)	1 (2.0)	6 (6.9)	3 (5.3)	1 (2.4)	1 (2.4	
F4	9 (3.2)	1 (2.0)	2 (2.3)	3 (5.3)	2 (4.8)	1 (2.4)	
Unknown	60	12	13	14	10	11	
Fibrosis (kPa), median (IQR)	5.0 (4.3–6.5)	5.6 (4.7–6.6)	4.8 (4.2–6.8)	5.1 (4.3–7.1)	4.7 (3.7–6.1)	4.9 (4.0–5.8)	0.25
Unknown E, n	60	12	13	14	10	11	
CAP Score (dB/m), mean (SD)	240 (52)	216 (55)	243 (50)	255 (50)	256 (50)	227 (41)	0.002
Unknown CAP, n	63	13	14	15	10	11	
ALT (U/L), median (IQR)	29 (21–45)	28 (20–42)	30 (22–48)	34 (26–48)	29 (21–47)	26 (20–33)	0.05
Unknown ALT, n	62	12	23	15	7	5	
AST (U/L), median (IQR)	22 (18–30)	24 (19–31)	20 (17–29)	25 (19–38)	22 (18–29)	20 (17–24)	0.01
Unknown AST, n	67	13	24	15	10	5	
AFP upper limit of normal (µg/L), median (IQR)	0.41 (0.29–0.67)	0.48 (0.29–0.86)	0.37 (0.24–0.49)	0.44 (0.30–0.61)	0.38 (0.29–0.71)	0.46 (0.32–1.0)	0.08
Unknown AFP, n ^3^	33	5	12	7	5	4	
Started antiviral therapy post baseline, n (%)							0.002
Yes	95 (28.0)	9 (14.3)	31 (31.0)	29 (40.9)	18 (34.6)	8 (15.1)	
No	244 (72.0)	54 (85.7)	69 (69.0)	42 (59.2)	34 (65.4)	45 (84.9)	

^1^ Chi-square and Fisher’s Exact tests (with Monte Carlo simulation [n = 1,000,000 samples] to estimate *p* values for large contingency tables) were used for categorical variables when assumptions were met. Kruskal-Wallis tests were used for statistical comparisons of continuous variables. A two-sided alpha level of 0.05 was used to determine statistical significance (*p* < 0.05). ^2^ Fibrosis stage was classified based on transient elastography scores (kPa). F0–1: 2–8 kPa, F2: 9–10 kPa, F3: 11–14 kPa, and F4: >14 kPa. ^3^ Values for AFP were normalized by dividing the values by the reference range cut-off due to changes in tests used over time (the upper limit of normal was 9 µg/L prior to 27 November 2019 and 7 µg/L after this date).

**Table 2:** Multivariable analysis of log-adjusted (A) HBV DNA (log10 IU/mL), (B) ALT (U/L), (C) AST (U/L), (D) Controlled Attenuation Parameter (CAP) Score (dB/m), and (E) Liver Fibrosis by transient elastography (kPa) over time according to treatments status during follow-up utilizing mixed effects regression models with an unstructured covariance structure, a linear trend, and interaction terms for time and genotype. The asterix (*) demotes statistical difference for the variable being assessed. The key finding of these analyses is that the variable ‘Time*Genotype’ which considers the influence of HBV genotype over time measured in days on the other variables in these multivariate models is consistently not statistically significant.

Variable		Estimate	95% CI	*p*
(A) Log-adjusted HBV DNA (log10 IU/mL)				
No Antiviral Treatment Cohort 365 days pre-baseline to 2635 days post (random intercept effects)				
HBV Genotype	A	0.1509	(−0.0002, 0.0003)	0.69
	B	0.5377	(−0.5998, 0.9016)	0.03 *
	D	−0.1182	(0.0514, 1.0240)	0.76
	E	0.2727	(−0.8917, 0.6553)	0.53
	C	Referent	-	-
Age		−0.0054	(−0.0181, 0.0073)	0.41
Race	Black	0.0203	(−0.7328, 0.7734)	0.96
	Asian	0.3573	(−0.3696, 1.0842)	0.33
	White	Referent	-	-
Gender	Female	−0.2301	(−0.5443, 0.0842)	0.15
	Male	Referent	-	-
HBeAg	Positive	3.3593	(2.5279, 4.1914)	<0.001 *
	Negative	Referent	-	-
Time*Genotype	A	−0.0002	(−0.0006, 0.0001)	0.15
	B	−0.0002	(−0.0005, 0.0001)	0.13
	D	−0.00002	(−0.00048, 0.00045)	0.94
	E	−0.00007	(−0.00042, 0.00028)	0.69
	C	Referent	-	-
Nucleos(t)ide Treatment Cohort 365 days pre-baseline to 365 days post (random intercept and slope effects)				
HBV Genotype	A	0.9055	(−2.9821, 4.7931)	0.64
	B	0.1641	(−0.7899, 1.1181)	0.73
	D	2.1049	(0.6661, 3.5437)	0.005 *
	E	−0.3470	(−4.1957, 3.5016)	0.86
	C	Referent	-	-
Age		−0.0058	(−0.0403, 0.0287)	0.74
Race	Black	1.1456	(−2.3559, 4.6471)	0.52
	Asian	1.3964	(−0.1783, 2.9711)	0.08
	White/Middle eastern	Referent	-	-
Gender	Female	0.2678	(−0.5166, 1.0522)	0.50
	Male	Referent	-	-
HBeAg	Positive	1.5427	(0.5512, 2.5342)	0.003 *
	Negative	Referent	-	-
Cirrhosis	Yes	−0.2963	(−1.4535, 0.8610)	0.61
	No	Referent	-	-
Time*Genotype	A	0.0040	(−0.0027, 0.0108)	0.23
	B	−0.0023	(−0.0069, 0.0024)	0.33
	D	0.0041	(−0.0011, 0.0093)	0.12
	E	0.0049	(−0.0016, 0.0115)	0.13
	C	Referent	-	-
(B) ALT (U/L)				
No Antiviral Treatment Cohort 365 days pre-baseline to 3000 days post (random intercept effects)				
HBV Genotype	A	−19.7162	(−35.9419, −3.4905)	0.02 *
	B	0.4957	(−11.0185, 12.0100)	0.93
	D	−40.4227	(−57.3385, −23.5069)	<0.001 *
	E	−34.3121	(−53.0935, −15.5307)	<0.001 *
	C	Referent	-	-
Age		−0.4057	(−0.6752, −0.1361)	0.003 *
Race	Black	−18.3939	(−34.8623, −1.9255)	0.03 *
	Asian	−34.5955	(−49.1318, −20.0592)	<0.001 *
	White	Referent	-	-
Gender	Female	−9.6095	(−16.1977, −3.0214)	0.004 *
	Male	Referent	-	-
HBeAg	Positive	6.7156	(−11.4222, 24.8535)	0.47
	Negative	Referent	-	-
Time*Genotype	A	0.003069	(−0.00826, 0.01440)	0.60
	B	−0.00203	(−0.01289, 0.008833)	0.71
	D	0.003643	(−0.01111, 0.01839)	0.63
	E	0.001265	(−0.01199, 0.01452)	0.85
	C	Referent	-	-
Log-adjusted ALT (log10 U/L)				
Nucleos(t)ide Treatment Cohort 365 days pre-baseline to 365 days post (random intercept and slope effects)				
HBV Genotype	A	−0.1079	(−0.7980, 0.5822)	0.76
	B	0.005804	(−0.1737, 0.1854)	0.95
	D	−0.01316	(−0.2721, 0.2458)	0.92
	E	−0.2232	(−0.9078, 0.4613)	0.52
	C	Referent	-	-
Age		−0.00381	(−0.00980, 0.002182)	0.21
Race	Black	0.04604	(−0.5697, 0.6618)	0.88
	Asian	−0.00053	(−0.2715, 0.2704)	0.997
	White	Referent	-	-
Gender	Female	−0.1287	(−0.2671, 0.009654)	0.07
	Male	Referent	-	-
HBeAg	Positive	−0.04479	(−0.2242, 0.1346)	0.62
	Negative	Referent	-	-
Cirrhosis	Yes	0.000534	(−0.1982, 0.1992)	0.996
	No	Referent	-	-
Time*Genotype	A	0.000010	(−0.00081, 0.000827)	0.98
	B	−0.00008	(−0.00061, 0.000437)	0.75
	D	−0.00013	(−0.00069, 0.000432)	0.65
	E	0.000666	(−0.00015, 0.001478)	0.11
	C	Referent	-	-
(C) AST (U/L)				
No Antiviral Treatment Cohort 365 days pre-baseline to 3000 days post (random intercept effects)				
HBV Genotype	A	−9.7813	(−17.9594, −1.6032)	0.02 *
	B	−1.5597	(−7.2674, 4.1479)	0.59
	D	−15.6206	(−24.0820, −7.1593)	0.0003 *
	E	−15.3909	(−24.8013, −5.9805)	0.001 *
	C	Referent	-	-
Age		−0.06855	(−0.2046, 0.06747)	0.32
Race	Black	−4.1607	(−12.4663, 4.1448)	0.33
	Asian	−12.3837	(−19.6938, −5.0736)	0.001 *
	White	Referent	-	-
Gender	Female	−2.9076	(−6.2309, 0.4156)	0.09
	Male	Referent	-	-
HBeAg	Positive	4.3911	(−4.5403, 13.3224)	0.33
	Negative	Referent	-	-
Time*Genotype	A	0.006122	(−0.00028, 0.01252)	0.06
	B	−0.00018	(−0.00590, 0.005540)	0.95
	D	0.001130	(−0.00644, 0.008705)	0.77
	E	0.001108	(−0.00616, 0.008375)	0.77
	C	Referent	-	-
Log-adjusted AST (log10 U/L)				
Nucleos(t)ide Treatment Cohort 365 days pre-baseline to 365 days post (random intercept effects)				
HBV Genotype	A	0.09063	(−0.4592, 0.6405)	0.74
	B	0.03623	(−0.1006, 0.1731)	0.60
	D	0.04719	(−0.1546, 0.2490)	0.64
	E	0.03965	(−0.5051, 0.5844)	0.89
	C	Referent	-	-
Age		0.000024	(−0.00496, 0.005006)	0.99
Race	Black	0.02645	(−0.4693, 0.5222)	0.92
	Asian	0.09306	(−0.1249, 0.3110)	0.40
	White	Referent	-	-
Gender	Female	−0.08023	(−0.1903, 0.02979)	0.15
	Male	Referent	-	-
HBeAg	Positive	−0.03670	(−0.1783, 0.1050)	0.61
	Negative	Referent	-	-
Cirrhosis	Yes	0.03342	(−0.1450, 0.2118)	0.71
	No	Referent	-	-
Time*Genotype	A	−0.00008	(−0.00072, 0.000555)	0.80
	B	−0.00011	(−0.00053, 0.000310)	0.61
	D	5.345 × 10^−6^	(−0.00046, 0.000472)	0.98
	E	−0.00034	(−0.00092, 0.000236)	0.25
	C	Referent	-	-
(D) CAP Score (dB/m)				
No Antiviral Treatment Cohort 365 days pre-baseline to 1500 days post (random intercept effects)				
HBV Genotype	A	−14.5561	(−44.3710, 15.2588)	0.34
	B	−0.7325	(−21.0382, 19.5732)	0.94
	D	−0.7322	(−33.7449, 32.2804)	0.97
	E	15.7653	(−18.3758, 49.9064)	0.36
	C	Referent	-	-
Age		0.7454	(0.2416, 1.2491)	0.004 *
Race	Black	−40.1231	(−68.6708, −11.5754)	0.006 *
	Asian	−13.8378	(−42.9904, 15.3149)	0.35
	White	Referent	-	-
Gender	Female	−13.4287	(−25.6344, −1.2231)	0.03 *
	Male	Referent	-	-
HBeAg	Positive	−23.0891	(−56.7218, 10.5436)	0.18
	Negative	Referent	-	-
Time*Genotype	A	0.01179	(−0.02702, 0.05061)	0.55
	B	−0.01641	(−0.05198, 0.01917)	0.36
	D	0.01907	(−0.02381, 0.06196)	0.38
	E	−0.03119	(−0.07201, 0.009636)	0.13
	C	Referent	-	-
Nucleos(t)ide Treatment Cohort 365 days pre-baseline to 1000 days post (random intercept effects)				
HBV Genotype	A	−65.8354	(−186.99, 55.3212)	0.28
	B	−36.8879	(−70.5333, −3.2425)	0.03 *
	D	−20.9892	(−77.2870, 35.3085)	0.46
	E	−56.1148	(−181.98, 69.7529)	0.38
	C	Referent	-	
Age		2.2586	(1.0942, 3.4230)	0.0003 *
Race	Black	34.7051	(−72.3055, 141.72)	0.52
	Asian	2.1161	(−60.5891, 64.8212)	0.95
	White/Middle eastern	Referent	-	-
Gender	Female	−15.6487	(−44.5327, 13.2353)	0.28
	Male	Referent	-	
HBeAg	Positive	18.2907	(−17.5021, 54.0834)	0.31
	Negative	Referent	-	
Cirrhosis	Yes	15.4410	(−23.7181, 54.6001)	0.43
	No	Referent	-	
Time*Genotype	A	0.08996	(−0.00922, 0.1891)	0.07
	B	0.01739	(−0.1230, 0.1577)	0.80
	D	0.002375	(−0.1095, 0.1143)	0.97
	E	0.06920	(−0.1664, 0.3048)	0.53
	C	Referent	-	-
(E) Liver Fibrosis (kPa)				
No Antiviral Treatment Cohort 365 days pre-baseline to 1700 days post (random intercept and slope effects; x-axis rescaled by dividing time by 10 to obtain convergence)				
HBV Genotype	A	−0.5795	(−1.8526, 0.6937)	0.37
	B	−0.2147	(−1.1487, 0.7194)	0.65
	D	−1.1898	(−2.5840, 0.2044)	0.09
	E	−1.3519	(−2.7874, 0.08361)	0.07
	C	Referent	-	-
Age		0.005415	(−0.01514, 0.02597)	0.60
Race	Black	0.4982	(−0.6937, 1.6901)	0.41
	Asian	−0.5027	(−1.6464, 0.6410)	0.39
	White/Middle eastern	Referent	-	-
Gender	Female	−0.3904	(−0.8706, 0.08975)	0.11
	Male	Referent	-	-
HBeAg	Positive	0.6700	(−0.5664, 1.9065)	0.28
	Negative	Referent	-	-
Time*Genotype	A	−0.00567	(−0.01903, 0.007694)	0.40
	B	−0.00514	(−0.01718, 0.006894)	0.40
	D	0.009461	(−0.00610, 0.02503)	0.23
	E	−0.00780	(−0.02175, 0.006145)	0.27
	C	Referent	-	-
Nucleos(t)ide Treatment Cohort 150 days pre-baseline to 1000 days post (random intercept effects)				
HBV Genotype	A	1.9698	(−2.7218, 6.6614)	0.41
	B	−2.7167	(−5.8594, 0.4259)	0.09
	D	1.1207	(−2.4235, 4.6649)	0.53
	E	1.2269	(−3.2905, 5.7442)	0.59
	C	Referent	-	-
Age		0.2611	(0.1619, 0.3602)	<0.0001 *
Gender	Female	−2.2149	(−4.8268, 0.3970)	0.10
	Male	Referent	-	-
HBeAg	Positive	4.0561	(0.9289, 7.1832)	0.01 *
	Negative	Referent	-	-
Time*Genotype	A	−0.00365	(−0.01262, 0.005322)	0.41
	B	−0.00184	(−0.01262, 0.008943)	0.73
	D	−0.00339	(−0.01366, 0.006874)	0.51
	E	0.002381	(−0.00595, 0.01071)	0.56
	C	Referent	-	-

## Data Availability

Data are available upon request from the corresponding author.

## Supplementary Materials

The following supporting information can be downloaded at: https://www.mdpi.com/article/10.3390/jcm12216807/s1, Table S1. HBV infection characteristics at baseline and at the time of HCC diagnosis for patients who were diagnosed with hepatocellular cancer (HCC) during follow-up. All HBV patients diagnosed with HCC were male and had no prior history of antiviral treatment when antiviral treatment was initiated at baseline. All HCC diagnoses were made after antiviral treatment was initiated.

## Appendix A

**Table A1:** HBV Genotype Subtypes. Subgenotype testing results were available and are reported for 229 of the 339 patients included in the study.

HBV Genotype Subtype ^1^	Number of Patients, n (%)
A1	14 (6.1)
A2	4 (1.8)
quasi-A3	8 (3.5)
A4	6 (2.6)
B1	3 (1.3)
B2	35 (15.3)
B3	8 (3.5)
B4	18 (7.9)
B5	1 (0.4)
C1	21 (9.2)
C2	21 (9.2)
C3	1 (0.4)
C5	1 (0.4)
C8-10	4 (1.8)
D1	13 (5.7)
D2	4 (1.8)
D2-GL	3 (1.3)
D3	3 (1.3)
D4	2 (0.9)
D5	2 (0.9)
D6	3 (1.3)
E	40 (17.5)
Unknown ^2^	14 (6.1)

^1^ The genotype subtype was estimated based on four phylogenetics methods using a short 284 nucleotides subgenomic HBsAg coding region. ^2^ The subgenotype could not be determined for 14 patients due to inconclusive results.

**Table A2:** Univariable analysis of log-adjusted (A) HBV DNA (log10 IU/mL), (B) ALT (U/L), (C) AST (U/L), (D) CAP Score (dB/m), and (E) E (kPa) over time according to treatment status during follow-up, using mixed effects regression with an unstructured covariance structure, a linear trend and a time interaction terms with time.

Variable		Estimate	95% CI		P
(A) Log-adjusted HBV DNA (log10 IU/mL)					
No Antiviral Treatment Cohort 365 days pre-baseline to 2635 days post (random intercept effects)					
HBV*Genotype	A	−0.2687	(−0.8395, 0.3021)		0.36
	B	0.5599	(0.01938, 1.1004)		0.04 *
	D	−0.4397	(−1.0594, 0.1799)		0.16
	E	−0.07787	(−0.6714, 0.5157)		0.80
	C	Referent	-		-
Time*Genotype	A	−0.00021	(−0.00055, 0.000136)		0.24
	B	−0.00011	(−0.00039, 0.000175)		0.45
	D	−0.00008	(−0.00048, 0.000325)		0.70
	E	−3.49 × 10^−7^	(−0.00036, 0.000362)		0.999
	C	Referent	-		-
Age		−0.00871	(−0.02215, 0.004727)		0.20
Time*Age		−3.91 × 10^−6^	(−0.00001, 4.625 × 10^−6^)		0.37
Race	Black	0.3830	(−0.2098, 0.9758)		0.21
	Asian	0.9369	(0.3654, 1.5084)		0.001 *
	White/Middle eastern	Referent	-		-
Time*Race	Black	−0.00016	(−0.00055, 0.000235)		0.43
	Asian	−0.00008	(−0.00043, 0.000278)		0.67
	White/Middle eastern	Referent	-		-
Gender	Female	−0.3469	(−0.7040, 0.01026)		0.06
	Male	Referent	-		-
Time*Gender	Female	0.000047	(−0.00016, 0.000258)		0.67
	Male	Referent	-		-
HBeAg	Positive	3.9872	(3.1931, 4.7813)		<0.001 *
	Negative	Referent	-		-
Time* HBeAg	Positive	−0.00035	(−0.00089, 0.000180)		0.19
	Negative	Referent	-		-
Liver cirrhosis	Yes	−0.3570	(−3.3762, 2.6621)		0.82
	No	Referent	-		-
Time*Cirrhosis	Yes	0.000586	(−0.00630, 0.007473)		0.87
	No	Referent	-		-
Nucleos(t)ide Treatment Cohort 365 days pre-baseline to 365 days post (random slope and intercept effects)					
HBV Genotype	A	0.3131	(−1.0628, 1.6890)		0.65
	B	−0.1855	(−1.1556, 0.7845)		0.71
	D	0.8909	(−0.1942, 1.9761)		0.11
	E	−0.9951	(−2.3756, 0.3853)		0.16
	C	Referent	-		-
Time*Genotype	A	0.003575	(−0.00256, 0.009710)		0.25
	B	−0.00192	(−0.00656, 0.002727)		0.41
	D	0.003912	(−0.00132, 0.009142)		0.14
	E	0.004633	(−0.00173, 0.01100)		0.15
	C	Referent	-		-
Age		−0.02037	(−0.04897, 0.008235)		0.16
Time*Age		−0.00020	(−0.00032, −0.00007)		0.002 *
Race	Black	0.5309	(−0.8446, 1.9063)		0.45
	Asian	0.6682	(−0.4903, 1.8266)		0.26
	White/Middle eastern	Referent	-		-
Time*Race	Black	0.003800	(−0.00236, 0.009966)		0.22
	Asian	−0.00150	(−0.00675, 0.003755)		0.57
	White/Middle eastern	Referent	-		-
Gender	Female	0.1961	(−0.6227, 1.0148)		0.64
	Male	Referent	-		-
Time*Gender	Female	−0.00059	(−0.00431, 0.003121)		0.75
	Male	Referent	-		-
HBeAg	Positive	1.7466	(0.9600, 2.5332)		<0.001 *
	Negative	Referent	-		-
Time*HBeAg	Positive	0.002683	(−0.00102, 0.006388)		0.15
	Negative	Referent	-		-
Liver cirrhosis	Yes	0.2173	(−0.9521, 1.3866)		0.71
	No	Referent	-		-
Time*Cirrhosis	Yes	−0.00517	(−0.01059, 0.000254)		0.06
	No	Referent	-		-
(B) ALT (U/L) (n = 3 cirrhotic patients excluded due to small sample size)					
No Antiviral Treatment Cohort 365 days pre-baseline to 3000 days post (random intercept effects)					
HBV Genotype	A	−5.5683	(−17.3707, 6.2342)		0.35
	B	−1.0695	(−12.2923, 10.1534)		0.85
	D	−9.0154	(−21.8919, 3.8611)		0.17
	E	−14.4553	(−27.0014, −1.9093)		0.02 *
	C	Referent	-		-
Time*Genotype	A	0.000868	(−0.01011, 0.01185)		0.88
	B	−0.00272	(−0.01306, 0.007611)		0.61
	D	0.000795	(−0.01182, 0.01341)		0.90
	E	−0.00028	(−0.01297, 0.01242)		0.97
	C	Referent	-		-
Age		−0.08881	(−0.3668, 0.1892)		0.53
Time*Age		−0.00011	(−0.00040, 0.000174)		0.44
Race	Black	−10.5320	(−23.6473, 2.5832)		0.12
	Asian	−5.6118	(−18.1782, 6.9547)		0.38
	White/Middle eastern	Referent	-		-
Time*Race	Black	0.002562	(−0.01170, 0.01682)		0.72
	Asian	0.000326	(−0.01319, 0.01384)		0.96
	White/Middle eastern	Referent	-		-
Gender	Female	−9.4869	(−16.8294, −2.1444)		0.01 *
	Male	Referent	-		-
Time*Gender	Female	0.003184	(−0.00381, 0.01018)		0.37
	Male	Referent	-		-
HBeAg	Positive	24.0903	(4.1640, 44.0165)		0.02 *
	Negative	Referent	-		-
Time*HBeAg	Positive	−0.00870	(−0.02924, 0.01184)		0.41
	Negative	Referent	-		-
Liver cirrhosis	Yes	41.4224	(−11.3433, 94.1881)		0.13
	No	Referent	-		-
Time*Cirrhosis	Yes	−0.00830	(−0.04003, 0.02342)		0.61
	No	Referent	-		-
Log-adjusted ALT (log10 U/mL)					
Nucleos(t)ide Treatment Cohort 365 days pre-baseline to 365 days post (random slope and intercept effects)					
HBV Genotype	A	−0.1534	(−0.3972, 0.09033)		0.22
	B	−0.01006	(−0.1847, 0.1646)		0.91
	D	0.09695	(−0.09663, 0.2905)		0.32
	E	−0.1471	(−0.3946, 0.1003)		0.24
	C	Referent	-		-
Time*Genotype	A	0.000011	(−0.00069, 0.000717)		0.98
	B	−0.00007	(−0.00057, 0.000437)		0.79
	D	−0.00017	(−0.00071, 0.000371)		0.53
	E	0.000764	(0.000015, 0.001513)		0.05 *
	C	Referent	-		-
Age		−0.00060	(−0.00569, 0.004487)		0.82
Time*Age		−2.83 × 10^−6^	(−0.00002, 0.000011)		0.69
Race	Black	−0.02931	(−0.2500, 0.1914)		0.79
	Asian	0.05517	(−0.1300, 0.2403)		0.56
	White/Middle eastern	Referent	-		-
Time*Race	Black	0.000117	(−0.00055, 0.000789)		0.73
	Asian	−0.00026	(−0.00079, 0.000276)		0.34
	White/Middle eastern	Referent	-		-
Gender	Female	−0.1290	(−0.2696, 0.01165)		0.07
	Male	Referent	-		-
Time*Gender	Female	−0.00020	(−0.00061, 0.000220)		0.35
	Male	Referent	-		-
HBeAg	Positive	−0.00843	(−0.1631, 0.1462)		0.91
	Negative	Referent	-		-
Time* HBeAg	Positive	−0.00002	(−0.00044, 0.000401)		0.93
	Negative	Referent	-		-
(C) AST (U/L)					
No Antiviral Treatment Cohort 365 days pre-baseline to 3000 days post (random intercept effects)					
HBV Genotype	A	−2.4263	(−7.9939, 3.1414)		0.39
	B	−1.9878	(−7.1858, 3.2103)		0.45
	D	−4.1509	(−10.1527, 1.8510)		0.18
	E	−5.9612	(−11.7672, −0.1553)		0.04 *
	C	Referent	-		-
Time*Genotype	A	0.001718	(−0.00254, 0.005978)		0.43
	B	−0.00177	(−0.00537, 0.001826)		0.33
	D	−0.00236	(−0.00649, 0.001768)		0.26
	E	−0.00160	(−0.00601, 0.002805)		0.48
	C	Referent	-		-
Age		−0.00893	(−0.1394, 0.1215)		0.89
Time*Age		0.000069	(−0.00004, 0.000174)		0.19
Race	Black	−2.7753	(−8.9335, 3.3830)		0.38
	Asian	−2.0412	(−7.9263, 3.8439)		0.50
	White/Middle eastern	Referent	-		-
Time*Race	Black	0.003011	(−0.00153, 0.007557)		0.19
	Asian	0.001797	(−0.00235, 0.005944)		0.40
	White/Middle eastern	Referent	-		-
Gender	Female	−3.5263	(−6.9848, −0.06789)		0.05*
	Male	Referent	-		-
Time*Gender	Female	0.000662	(−0.00202, 0.003343)		0.63
	Male	Referent	-		-
HBVeAg	Positive	10.2458	(0.9892, 19.5025)		0.03 *
	Negative	Referent	-		-
Time*HBVeAg	Positive	−0.00457	(−0.01106, 0.001913)		0.17
	Negative	Referent	-		-
Log-adjusted AST (log10 U/mL)					
Nucleos(t)ide Treatment Cohort 365 days pre-baseline to 365 days post (random intercept effects)					
HBV Genotype	A	−0.08093	(−0.2918, 0.1300)		0.45
	B	0.02676	(−0.1244, 0.1779)		0.73
	D	0.07900	(−0.09039, 0.2484)		0.36
	E	−0.06335	(−0.2746, 0.1479)		0.55
	C	Referent	-		-
Time*Genotype	A	0.000025	(−0.00061, 0.000664)		0.94
	B	−0.00009	(−0.00056, 0.000380)		0.71
	D	0.000021	(−0.00050, 0.000543)		0.94
	E	0.000016	(−0.00053, 0.000564)		0.95
	C	Referent	-		-
Age		0.002953	(−0.00140, 0.007302)		0.18
Time*Age		2.055 × 10^−6^	(−0.00001, 0.000015)		0.76
Race	Black	0.05925	(−0.1088, 0.2273)		0.49
	Asian	0.08827	(−0.05396, 0.2305)		0.22
	White/Middle eastern	Referent	-		-
Time*Race	Black	−0.00021	(−0.00080, 0.000382)		0.49
	Asian	−0.00019	(−0.00071, 0.000333)		0.48
	White/Middle eastern	Referent	-		-
Gender	Female	−0.1012	(−0.2216, 0.01918)		0.10
	Male	Referent	-		-
Time*Gender	Female	−0.00021	(−0.00059, 0.000166)		0.27
	Male	Referent	-		-
HBeAg	Positive	−0.03775	(−0.1690, 0.09353)		0.57
	Negative	Referent	-		-
Time* HBeAg	Positive	0.000129	(−0.00021, 0.000465)		0.45
	Negative	Referent	-		-
Liver cirrhosis	Yes	0.04413	(−0.1505, 0.2387)		0.66
	No	Referent	-		-
Time*Cirrhosis	Yes	−0.00018	(−0.00074, 0.000389)		0.54
	No	Referent	-		-
(D) CAP Score (dB/m) (n = 1 cirrhotic patient excluded from analysis)					
No Antiviral Treatment Cohort 365 days pre-baseline to 2000 days post (random intercept and slope effects; *x*-axis rescaled by dividing time by 10 to obtain convergence ^1^)					
Genotype ^1^	A	−35.1238	(−56.3335, −13.9142)		0.001 *
	B	−3.3852	(−23.5331, 16.7626)		0.74
	D	2.9277	(−20.3939, 26.2492)		0.81
	E	−20.1950	(−41.9836, 1.5935)		0.07
	C	Referent	-		-
Time*Genotype	A	0.2053	(−0.1119, 0.5226)		0.20
	B	−0.06159	(−0.3434, 0.2203)		0.66
	D	0.09995	(−0.2489, 0.4488)		0.57
	E	−0.1662	(−0.5190, 0.1865)		0.35
	C	Referent	-		-
Age ^1^		0.9291	(0.4431, 1.4151)		<0.001 *
Time*Age		0.001855	(−0.00639, 0.01010)		0.65
Race ^1,2^	Black	−39.5702	(−61.3150, −17.8253)		<0.001 *
	Asian	−12.3440	(−33.4274, 8.7395)		0.25
	White/Middle eastern	Referent	-		-
Time*Race	Black	−0.1821	(−0.5131, 0.1489)		0.28
	Asian	−0.3247	(−0.6319, −0.01764)		0.04 *
	White/Middle eastern	Referent	-		-
Gender	Female	−13.0896	(−26.2086, 0.02938)		0.05 *
	Male	Referent	-		-
Time*Gender	Female	−0.02268	(−0.2149, 0.1696)		0.81
	Male	Referent	-		-
HBeAg ^2^	Positive	−15.9516	(−52.5582, 20.6550)		0.39
	Negative	Referent	-		-
Time* HBeAg	Positive	−0.01127	(−0.05921, 0.03666)		0.64
	Negative	Referent	-		-
Nucleos(t)ide Treatment Cohort 365 days pre-baseline to 365 days post (random intercept and slope effects, except where specified otherwise ^2^)					
HBV Genotype	A	−33.6937	(−83.6431, 16.2558)		0.18
	B	−35.9316	(−77.1145, 5.2513)		0.09
	D	−34.4497	(−80.2472, 11.3478)		0.14
	E	−69.2815	(−121.88, −16.6837)		0.01 *
	C	Referent	-		-
Time*Genotype	A	0.1049	(−0.2618, 0.4717)		0.53
	B	0.07445	(−0.2787, 0.4275)		0.66
	D	−0.04099	(−0.4325, 0.3505)		0.82
	E	0.1853	(−0.3239, 0.6946)		0.37
	C	Referent	-		-
Age ^2^		1.4483	(0.5051, 2.3915)		0.003 *
Time*Age		−0.00341	(−0.01246, 0.005646)		0.45
Race ^2^	Black	−22.5802	(−74.9517, 29.7913)		0.39
	Asian	17.2820	(−29.8917, 64.4557)		0.47
	White/Middle eastern	Referent	-		-
Time*Race	Black	0.2282	(−0.1608, 0.6172)		0.25
	Asian	0.2771	(−0.09130, 0.6454)		0.14
	White/Middle eastern	Referent	-		-
Gender	Female	−14.6130	(−47.1468, 17.9207)		0.37
	Male	Referent	-		-
Time*Gender	Female	−0.03639	(−0.2989, 0.2261)		0.77
	Male	Referent	-		-
HBeAg ^2^	Positive	10.2724	(−20.4380, 40.9828)		0.51
	Negative	Referent	-		-
Time* HBeAg	Positive	0.09380	(−0.1052, 0.2928)		0.35
	Negative	Referent	-		-
Liver Cirrhosis	Yes	47.8104	(−139.32, 234.94)		0.23
	No	Referent	-		-
Time*Cirrhosis	Yes	0.03897	(−0.6534, 0.7314)		0.91
	No	Referent	-		-
(E) Liver Fibrosis (kPa)					
No Antiviral Treatment Cohort 365 days pre-baseline to 1700 days post (random intercept and slope effects; *x*-axis was rescaled by dividing number of days from baseline date by 10)					
Genotype	A	0.3459	(−0.5593, 1.2512)		0.45
	B	−0.03412	(−0.8985, 0.8303)		0.94
	D	−0.6787	(−1.6772, 0.3199)		0.18
	E	−0.4021	(−1.3350, 0.5308)		0.40
	C	Referent	-		-
Time*Genotype	A	−0.00836	(−0.02112, 0.004393)		0.20
	B	−0.00724	(−0.01860, 0.004123)		0.21
	D	0.007210	(−0.00734, 0.02176)		0.33
	E	−0.00967	(−0.02300, 0.003671)		0.15
	C	Referent	-		-
Age		0.01062	(−0.01023, 0.03148)		0.32
Time*Age		−0.00011	(−0.00042, 0.000207)		0.50
Race	Black	0.2559	(−0.7030, 1.2149)		0.60
	Asian	0.07160	(−0.8603, 1.0035)		0.88
	White/Middle eastern	Referent	-		-
Time*Race	Black	−0.01021	(−0.02327, 0.002845)		0.12
	Asian	−0.00657	(−0.01877, 0.005620)		0.29
	White/Middle eastern	Referent	-		-
Gender	Female	−0.3269	(−0.8792, 0.2254)		0.25
	Male	Referent	-		-
Time*Gender	Female	0.000318	(−0.00718, 0.007819)		0.93
	Male	Referent	-		-
HBeAg	Positive	0.1930	(−1.2726, 1.6587)		0.80
	Negative	Referent	-		-
Time*HBeAg	Positive	0.007377	(−0.01082, 0.02557)		0.42
	Negative	Referent	-		-
Nucleos(t)ide Treatment Cohort 150 days pre-baseline to 800 days post (random intercept effects)					
HBV Genotype	A	0.1492	(−5.1524, 5.4508)		0.96
	B	−2.1196	(−2.9971, 5.2578)		0.24
	D	1.1303	(−2.9971, 5.2578)		0.59
	E	−2.2205	(−7.2091, 2.7681)		0.38
	C	Referent	-		-
Time*Genotype	A	−0.01491	(−0.02471, −0.00510)		0.005 *
	B	−0.01032	(−0.01884, −0.00179)		0.02 *
	D	−0.00742	(−0.01948, 0.004630)		0.21
	E	−0.00028	(−0.01481, 0.01425)		0.97
	C	Referent	-		-
Age		0.1796	(0.08964, 0.2696)		<0.001 *
Time*Age		−0.00003	(−0.00034, 0.000290)		0.87
Race	Black	1.7520	(−2.9654, 6.4695)		0.46
	Asian	1.8647	(−2.0685, 5.7980)		0.35
	White/Middle eastern	Referent	-		-
Time*Race	Black	−0.01202	(−0.02437, 0.000333)		0.06
	Asian	−0.00903	(−0.01983, 0.001774)		0.10
	White/Middle eastern	Referent	-		-
Gender	Female	−3.3599	(−6.1776, −0.5422)		0.02 *
	Male	Referent	-		-
Time*Gender	Female	0.000204	(−0.00969, 0.01009)		0.97
	Male	Referent	-		-
HBeAg	Positive	0.8919	(−2.4538, 4.2376)		0.60
	Negative	Referent	-		-
Time*HBeAg	Positive	−0.00390	(−0.01478, 0.006984)		0.47
	Negative	Referent	-		-
Liver Cirrhosis	Yes	6.5899	(3.1923, 9.9876)		<0.001 *
	No	Referent	-		-
Time*Cirrhosis	Yes	−0.00195	(−0.01404, 0.01014)		0.75
	No	Referent	-		-

^1^ The *x*-axis rescaled by dividing the number of days from baseline date by 10 (and each time unit change is equal to a change of 10 days). To obtain a non-adjusted estimate (where one day corresponds to a single time unit), divide the reported estimates and confidence interval limits in the table by 10. ^2^ Fitted models include random intercepts effects (and not random slope effects). * An alpha level cut-off of 0.05 was used to determine significance.
