# Supplementary material for: Hepatitis B Virus Genotype Influence on Virological and Enzymatic Measures over Time—A Retrospective Longitudinal Cohort Study
Source: J Clin Med. 2023 Oct 27;12(21):6807. doi: 10.3390/jcm12216807 (PMC10649073; doi:10.3390/jcm12216807)
Supplement: Supplementary file 1 [file jcm-12-06807-s001.zip › jcm-2639403-supplementary.pdf]

Table S1. HBV infection characteristics at baseline and at the time of HCC diagnosis for patients who were diagnosed with hepatocellular cancer (HCC) during follow-up [1].

Variable	Patient			
	**1**	**2**	**3**	**4**
HBV Genotype	B	D	B	E
Antiviral therapy	Tenofovir Disoproxil Fumarate	Tenofovir Disoproxil Fumarate	Tenofovir Disoproxil Fumarate	Lamivudine
Antiviral therapy outcome	HBV DNA below limit of detection following treatment initiation. Remains on therapy.	HBV DNA below limit of detection following treatment initiation. Remains on therapy.	HBV DNA below limit of detection following treatment initiation. Remains on therapy.	HBV DNA below limit of detection following treatment initiation. Remains on therapy.
At Baseline:				
Age	57	68	62	31
HBV DNA (IU/mL)	1.29×10^6	1.12×10^4	8.44×10^3	1.25×10^5
ALT (U/L)	44	41	50	25
AST (U/L)	31	28	48	22
Fibrosis (kPa)	17.3	10.5	4.3	15.4
CAP Score (dB/m)	302	288	192	249
At HCC Diagnosis:				
Age	60	70	62	34
HBV DNA (IU/mL)	<20	4.76×10^1	9.89×10^2	<20
ALT (U/L)	62	151	207	25
AST (U/L)	76	153	231	17
Fibrosis (kPa)	-	5.9	-	-
CAP Score (dB/m)	-	291	-	-

[1] All HBV patients diagnosed with HCC were male and had no prior history of antiviral treatment when antiviral treatment was initiated at baseline. All HCC diagnoses were made after antiviral treatment was initiated.
